# High tibial osteotomy for medial meniscus posterior root tears in knees with moderate varus alignment can achieve favorable clinical outcomes

**DOI:** 10.1186/s40634-022-00504-9

**Published:** 2022-07-07

**Authors:** Junya Itou, Umito Kuwashima, Masafumi Itoh, Ken Okazaki

**Affiliations:** Department of Orthopaedic Surgery, Tokyo Women’s Medical University, 8-1 Kawada-cho, Shinjuku-ku, Tokyo, 162-8666 Japan

**Keywords:** Medial meniscus posterior root tear, High tibial osteotomy, Patient-reported outcome measures, Moderate varus alignment

## Abstract

**Purpose:**

Favorable clinical results have been reported following high tibial osteotomy (HTO) for medial meniscus posterior root tear (MMPRT) in knees with varus alignment. However, the effect on the preoperative neutral alignment of the knee is not known. This study sought to evaluate the clinical outcomes of medial open-wedge HTO for MMPRT with neutral alignment.

**Methods:**

We retrospectively reviewed 119 medial open-wedge HTOs and analyzed 22 knees with MMPRT. The knees were divided according to the preoperative hip-knee-ankle angle into a moderate varus alignment group (≤4° of varus alignment) and a varus alignment group (> 4° of varus alignment). The Knee Injury and Osteoarthritis Outcome Score (KOOS) and Forgotten Joint Score-12 (FJS-12) values were evaluated preoperatively and at the latest follow-up. The healing status of MMPRT at the time of second-look arthroscopy, performed at a mean of 15.4 ± 4.2 months, was compared with that after the primary HTO.

**Results:**

There were 11 knees in the moderate varus alignment group and 11 in the varus alignment group. In terms of perioperative patient-reported outcome measures, there was no significant difference in the preoperative or postoperative KOOS subscale score or FJS-12 score between the moderate varus and varus alignment groups. The healing rate was significantly higher in the moderate varus alignment group.

**Conclusion:**

Favorable clinical results were obtained by medial open-wedge HTO in knees with MMPRT and moderate varus alignment in the short term. Surgeons should consider the indications for medial open-wedge HTO, even with moderate varus alignment, when planning treatment for MMPRT with persistent knee pain.

**Level of evidence:**

IV

**Supplementary Information:**

The online version contains supplementary material available at 10.1186/s40634-022-00504-9.

## Introduction

Medial meniscus posterior root tear (MMPRT) is increasingly recognized to accelerate degenerative changes in the knee [4, 21]. For knees with MMPRT and varus malalignment, favorable clinical results following medial open-wedge high tibial osteotomy (HTO) have been reported [20]. There seems to be a consensus that more than 4° or 5° of varus alignment preoperatively is an indication for HTO. Jing et al. recommended medial open-wedge HTO for MMPRT and varus alignment of > 4° [8] whereas Kim et al. recommended > 5° of varus as an indication for this surgery [11].

For patients with a well-aligned knee, favorable outcomes after transtibial pullout repair of MMPRT have been reported [1, 3]. However, this arthroscopic procedure is technically demanding and time-consuming [24]. Kim et al. reported that 35.7% of patients who underwent transtibial pullout repair of the medial meniscus showed abnormal fixation strength and progression of arthrosis on second-look arthroscopic examination [12]. Furthermore, Moon et al. reported that medial meniscus extrusion (MME) increased from 3.6 mm preoperatively to 5.0 mm postoperatively, which was contrary to expectations [18]. MME sometimes coexists with MMPRT even in knees with neutral alignment [15]. Thus, there are several challenges with transtibial pullout repair of MMPRT. Meanwhile, medial open-wedge HTO is a well-established surgical procedure and is familiar to knee surgeons. Na et al. recently reported that medial open-wedge HTO was useful for patients with osteoarthritis and mild varus alignment of < 4° [19]. However, the effect of medial open-wedge HTO on MMPRT in well-aligned knees is still unknown.

The purpose of this study was to evaluate the clinical outcomes of medial open-wedge HTO for MMPRT in well-aligned knees. The hypothesis was that medial open-wedge HTO for MMPRT in a well-aligned knee would have an outcome similar to that of medial open-wedge HTO for MMPRT in a knee with varus alignment.

## Materials and methods

### Patient selection and study design

This retrospective study was approved by our institutional ethics committee (approval number: 4578). From April 2017 to October 2020, a total of 119 medial open-wedge HTOs were performed for varus knees with osteoarthritis (OA) or osteonecrosis at our institution. All of the patients still complained of medial knee pain after at least 3 months of conservative treatment including physical therapy, pain medications, and weight loss. In total, 22 knees of 20 patients (4 men, 16 women) diagnosed with MMPRT were included in the study. Mean age at surgery was 57.1 ± 8.3 years and mean body mass index (calculated as kg/m^2^) was 26.1 ± 4.8. MMPRT was diagnosed based on characteristic findings on magnetic resonance images (cleft/truncation sign, ghost/white meniscus sign, radial tear sign, giraffe neck sign) [4, 9, 21] that were confirmed by arthroscopy.

### Surgical technique and postoperative rehabilitation

**Fig. 1:**
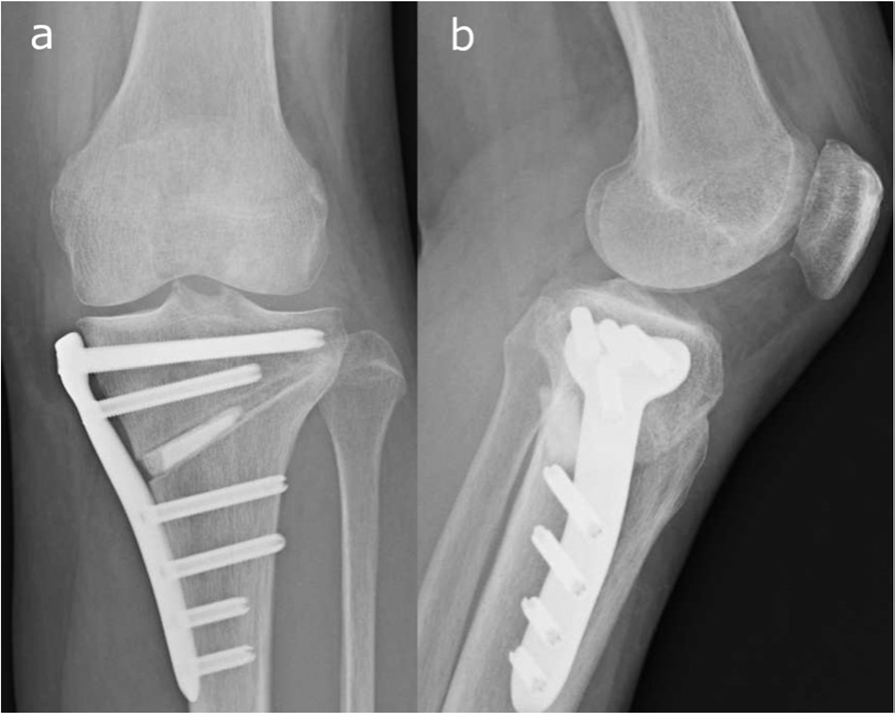
Radiographs after medial open-wedge HTO. **A** Anteroposterior radiograph. **B** Lateral radiograph

**Fig. 2:**
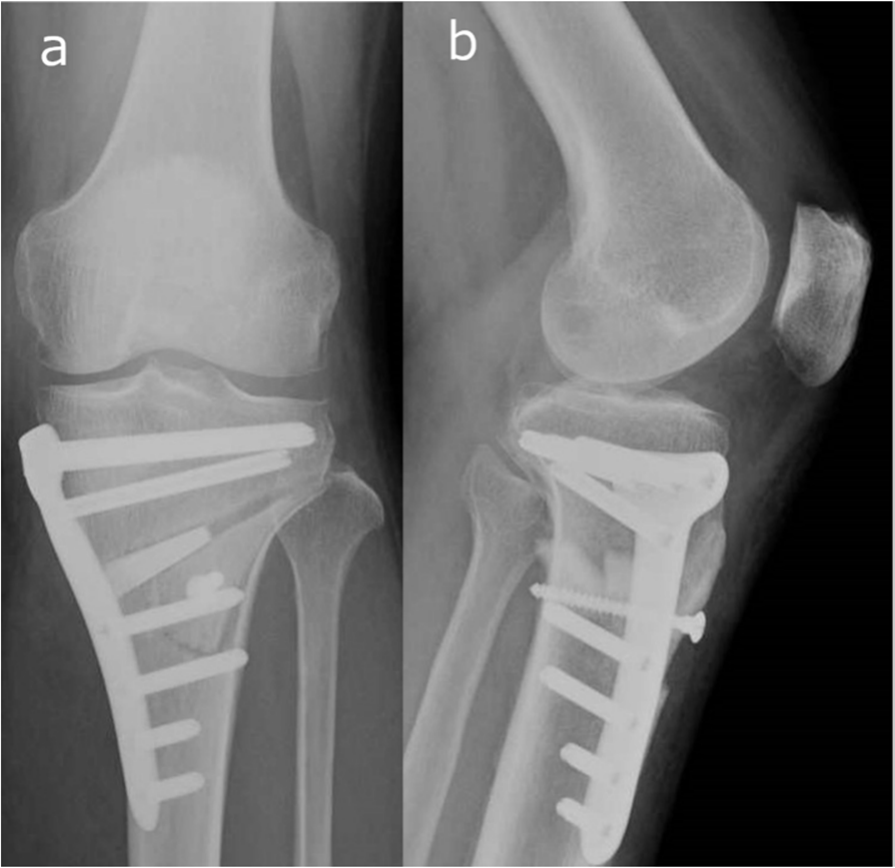
Radiographs after medial open-wedge DTO. **A** Anteroposterior radiograph. **B** Lateral radiograph

The surgical procedures were performed by any of four specialist knee surgeons, with attention paid to consistency in surgical techniques used and intraoperative management across cases. HTO was performed by the medial open-wedge HTO (Fig. 1) or medial open-wedge distal tibial tuberosity osteotomy (DTO) (Fig. 2) method using a long locking plate (TriS, Olympus Terumo Biomaterials, Tokyo, Japan) [2, 7, 12]. In open-wedge DTO, the tibial tuberosity remains attached to the proximal tibia, and bi- cortical screw fixation from the tuberosity to the posterior tibia was performed to support the descending osteotomy [2]. The surgical procedure was chosen by the surgeon based on the patellofemoral joint problem. Patients with symptomatic patellofemoral joint space narrowing and/or osteophytes were excluded [10]. In all cases, artificial bone (OSferion 60, Olympus Terumo Biomaterials, Tokyo, Japan) was inserted into the osteotomy site. The correction angle was determined by aiming for a postoperative weight-bearing axis at a point 62.5% lateral to the transverse diameter of the tibial plateau on double-limb standing full-length lower extremity radiographs [6, 11].

Concurrent arthroscopic resection or repair using an all-inside meniscal suture device (TrueSpan, DePuy Mitek, Inc., Raynham, MA; Fast-Fix 360, Smith & Nephew Endoscopy, Andover, MA) was performed for MMPRT at the discretion of the surgeon. None of the knees underwent a transtibial pullout repair for MMPRT.

All patients were allowed to perform toe­touch weight bearing using double crutches and range-of-motion exercises immediately after surgery. Full weightbearing was permitted 3 weeks later.

### Radiological parameters and PROMs

The hip-knee-ankle (HKA) angle was measured preoperatively and postoperatively on double-limb standing full-length lower extremity radiographs. The knees were divided according to the preoperative HKA angle into a moderate varus alignment group (≤4° of varus alignment) and a varus alignment group (> 4° of varus alignment) [8]. Degenerative change was recorded according to the Kellgren-Lawrence OA grade on preoperative plain radiographs.

MME was defined as meniscal displacement of ≥3 mm and measured from the superior medial edge of the tibial plateau to the circumference of the medial meniscus body on preoperative coronal magnetic resonance images [4, 12, 15].

Intraobserver reliability of measurements was assessed using the intraclass correlation coefficient (ICC). Measurements were repeated after a 2-week interval to evaluate the ICC for the radiological parameter (HKA angle) in all 22 knees. The ICC for intraobserver agreement of the radiological HKA angle was 0.92.

Patient data, including age, sex, preoperative body mass index, and affected side were obtained from the medical records.

To evaluate patient-reported outcome measures (PROMs), patients were asked to complete the Knee Injury and Osteoarthritis Outcome Score (KOOS) and Forgotten Joint Score-12 (FJS-12) preoperatively and postoperatively by the attending surgeon. Postoperative PROMs were measured at the final outpatient visit more than 1 year after surgery.

The minimum clinically important difference (MCID) for preoperative and postoperative changes in the KOOS subscale scores following HTO was as follows: pain, 15.4; symptoms, 15.1; activities of daily living, 17.0; sport, 11.2; and quality of life 16.5 [7].

### Second-look arthroscopic evaluation

Second-look arthroscopy was performed to remove the HTO plate and evaluate the status of the MMPRT at a mean of 15.4 ± 4.2 months after the primary HTO. The status of the MMPRT at the time of second-look arthroscopy was compared with that at the time of the primary HTO recorded in the medical records. Healing of the MMPRT was classified as complete, partial, or no healing [10, 17]. Complete healing was defined as meniscal continuity with no cleft, no lifting on probing, and normal meniscal tension. No healing was defined as no meniscal continuity, no tension on probing, and no evidence of healing [13, 17]. Partial healing was defined as intermediate between complete and no healing.

### Statistical analysis

The chi-squared and Cochran-Armitage tests were used to determine the statistical significance of between-group differences in categorical variables and the Wilcoxon signed-rank test to evaluate the significance of between-group differences in continuous variables. All statistical analyses were performed using JMP software version 16 (SAS Institute Inc., Cary, NC, USA) and G*Power version 3.1.9.7 (Universität Kiel, Kiel, Germany). A *p*-value < 0.05 was considered statistically significant. The sample size was calculated with a power of 81% and α of 0.05 [13]. The required sample size was 18 and 22 patients were included in this study.

## Results

The 22 knees were divided according to the preoperative HKA angle into a moderate varus alignment group (*n* = 11) and a varus alignment group (n = 11). There was no significant difference in preoperative age, sex ratio, body mass index, diagnosis, or surgical procedure performed between the two groups (Table 1). However, the Kellgren-Lawrence OA grade indicated that there were significantly more cases of early-stage OA in the moderate varus alignment group (*p* = 0.03). The mean HKA angle was 2.4 ± 1.3° preoperatively and − 3.7 ± 0.9° postoperatively in the moderate varus alignment group and 5.8 ± 1.6° and − 2.7 ± 2.2°, respectively, in the varus alignment group (Table 1). Mean preoperative MME was 4.3 ± 0.8 mm in the moderate varus alignment group and 4.9 ± 1.1 mm in the varus alignment group (*p* = 0.17).

**Table 1:** Patient characteristics

	Moderate varus alignment (*n* = 11)	Varus alignment (*n* = 11)	*p-*value
Age, years, mean ± SD	54.8 ± 6.7	59.4 ± 9.4	0.23
Male sex, n (%)	3 (27.2)	2 (18.1)	1.00
K/L grade, 1,2,3,4	6,4,1,0	2,2,6,0	0.03
Body mass index, mean ± SD	24.9 ± 2.9	27.1 ± 6.4	0.57
Right side affected, n (%)	8 (72.7)	4 (36.3)	0.19
Diagnosis, OA, n (%)	11 (100)	10 (90.9)	1.00
Surgical procedure (OWHTO, %)	9 (81.8)	8 (72.7)	1.00
Pre-HKA angle	2.4 ± 1.3°	5.8 ± 1.6°	< 0.0001
Post-HKA angle	−3.7 ± 0.9°	−2.7 ± 2.2°	0.18
ΔHKA	6.1 ± 1.8°	8.5 ± 3.1°	0.10
MME (mm)	4.3 ± 0.8	4.9 ± 1.1	0.17

*BMI* Body mass index, *HKA* Hip-knee-ankle, *K/L grade*, Kellgren-Lawrence *OA* grade, *MME* Medial meniscus extrusion, *OA* Osteoarthritis, *OWHTO* Open-wedge high tibial osteotomy, *Post* Postoperative, *Pre* Preoperative; *SD* Standard deviation

On assessment of perioperative PROMs, there was no significant difference in the preoperative or postoperative FJS-12 or KOOS subscale scores between the moderate varus and varus alignment groups (Tables 2 and 3). At a mean postoperative follow-up of 23.5 months, all PROMs showed significant improvement. In the moderate varus alignment group, the numbers of knees for which the MCID was exceeded were as follows: pain, 8; symptoms, 8; activities of daily living, 6; sport, 10; and quality of life, 10. In the varus alignment group, the numbers of knees for which the MCID was exceeded were as follows: pain, 9; symptoms, 8; activities of daily living, 9; sport, 10; and quality of life, 9.

**Table 2:** Comparison of preoperative value for each patient-reported outcome measure between the moderate varus alignment group and the varus alignment group

Alignment before surgery	FJS-12	KOOS (Pain)	KOOS (Symptoms)	KOOS (ADL)	KOOS (Sports)	KOOS (QoL)
Moderate varus	14.2 ± 13.0	55.2 ± 18.3	51.3 ± 20.2	67.1 ± 14.7	26.8 ± 25.6	27.8 ± 23.2
Varus	27.4 ± 20.6	54.8 ± 20.1	58.8 ± 19.6	66.8 ± 18.1	36.3 ± 32.9	40.3 ± 24.2
*p*-value	0.09	0.89	0.59	0.94	0.64	0.18

Data are presented as the mean and standard deviation. *ADL* Activities of daily living, *FJS-12* Forgotten Joint Score, *KOOS* Knee Injury and Osteoarthritis Outcome Score, *QoL* Quality of life

**Table 3:** Comparison of postoperative value for each patient-reported outcome measure between the moderate varus alignment group and the varus alignment group

Alignment after surgery	FJS-12	KOOS (Pain)	KOOS (Symptoms)	KOOS (ADL)	KOOS (Sports)	KOOS (QoL)
Moderate varus	57.5 ± 25.6	82.8 ± 18.9	82.4 ± 14.5	88.1 ± 14.2	69.0 ± 23.1	72.7 ± 18.5
Varus	65.3 ± 25.3	88.8 ± 12.8	90.2 ± 11.1	90.5 ± 11.6	72.2 ± 26.3	76.1 ± 17.4
*p*-value	0.30	0.35	0.12	0.64	0.66	0.55

Data are presented as the mean and standard deviation. *ADL* Activities of daily living, *FJS-12* Forgotten Joint Score, *KOOS* Knee Injury and Osteoarthritis Outcome Score, *QoL* Quality of life

MMPRT was repaired using an all-inside meniscal suture device in 5 of the 22 knees. During second-look arthroscopy, complete healing of the MMPRT was observed in 20.0% (1/5) of knees in the repaired group and in 5.8% (1/17) of knees in the arthroscopic resection group. Similarly, partial healing of the MMPRT was observed in 60.0% (3/5) of knees in the repaired group and in 41.1% (7/17) of those in the arthroscopic resection group. Furthermore, no healing of the MMPRT was observed in 20.0% (1/5) of knees in the repaired group and in 52.9% (9/17) of those in the arthroscopic resection group (Table 4). In terms of preoperative alignment, there was no obvious difference in the proportion of knees that were repaired in either alignment group. However, the healing rate was significantly higher in the moderate varus alignment group (*p* = 0.04) (Table 4).

**Table 4:** Comparison of healing status of MMPRT, preoperative alignment, and concurrent arthroscopic procedure

	Moderate varus alignment	Varus alignment	*p*-value	Repaired	Resection	*p*-value
**Healing status**			0.04			0.43
Complete, n (%)	2 (18.2)	0 (0)		1 (20)	1 (5.9)	
Partial, n (%)	7 (63.6)	3 (27.3)		3 (60)	7 (41.2)	
No healing, n (%)	2 (18.2)	8 (72.7)		1 (20)	9 (52.9)	
**Concurrent arthroscopic procedure**			0.84			
Repaired, n (%)	3 (27.3)	2 (18.2)				
Resection, n (%)	8 (72.7)	9 (81.8)				

Chi-squared and Cochran-Armitage tests were used

## Discussion

The most important finding in this study was that the clinical results of medial open-wedge HTO for MMPRT in knees with moderate varus alignment were favorable after a mean short-term follow-up of 23.5 months. Even in knees with moderate varus alignment, medial open-wedge HTO could achieve clinical improvement that exceeded the MCID for KOOS in many cases. Our findings highlight the need for surgeons to consider the indications for medial open-wedge HTO even in knees with moderate varus alignment when planning treatment for MMPRT.

Surgical treatment of MMPRT remains controversial [21]. The most common treatments are partial meniscectomy and transtibial pullout or suture anchor repair [9, 20]. However, in the varus knee, medial open-wedge HTO can off-load the medial compartment of the tibiofemoral joint with favorable clinical outcomes [8]. Ahn et al. considered varus alignment to be a poor prognostic factor in pull-out repair of MMPRT [1]. Several studies have reported that concurrent arthroscopic meniscal repair during medial open-wedge HTO has little benefit in terms of the clinical outcomes of treatment for MMPRT [8, 10, 14]. Similarly, favorable clinical outcomes have been reported for medial open-wedge HTO without any attempt at meniscal treatment [16, 17]. However, it is unclear whether a relatively small angle correction by medial open-wedge HTO has a similarly favorable outcome in knees with MMPRT and moderate varus alignment. An important feature of this study was that it identified MMPRT with moderate varus knee alignment as a possible new indication for medial open-wedge HTO. On second-look arthroscopy, there was no significant difference in the MMPRT healing rate between the repaired group and the arthroscopic debridement group. Further research may be needed to determine whether a concurrent arthroscopic repair is beneficial.

The postoperative target alignment of HTO is also controversial. In this study, the mechanical axis was set to pass through 62.5% of the tibial plateau. Several studies have reported favorable clinical outcomes with similar postoperative alignment [5, 10, 11, 16, 17]. Na et al. reported that the functional outcomes were comparable between knees with mild varus (≤4° of varus alignment) preoperatively and knees with greater varus [19]. In their study, postoperative alignment was corrected into more valgus in the greater varus group than in the mild varus group. Further research is needed to determine whether the target alignment should be the same in knees with moderate varus alignment as in those with varus alignment before surgery.

We found a significant difference in healing status of the MMPRT between our two alignment groups (Table 4). In knees with varus alignment, the tibial joint surface was steeply tilted and the MME may have been advanced [21]. A biomechanical study by Willinger et al. [23] found that the MME was significantly greater in knees with varus alignment than in those with moderate varus alignment. It was widely known that MMPRT correlates with MME [4, 22]. Kim et al. [13] reported that the MME was 3.0 ± 0.7 mm preoperatively and 3.1 ± 0.7 mm postoperatively in patients with MMPRT who underwent arthroscopic meniscal suture repair concomitant with medial open-wedge HTO. Furthermore, Lee et al. [17] found no statistically significant difference between the preoperative and postoperative MME in patients who underwent medial open-wedge HTO, regardless of whether or not the meniscus was repaired. Therefore, the effect of HTO on MME was not considered to be significant, and preoperative alignment may have affected the MMPRT healing rate.

This study has several limitations. First, the retrospective nature of the study raises the possibility of selection bias. Furthermore, the moderate varus alignment group contained more cases of early-stage OA. It is possible that elective unicompartmental knee arthroplasty was performed for advanced OA. Second, the sample size was relatively small and the follow-up duration was short. Medium-term to long-term follow-up may be necessary to further evaluate the outcomes of medial open-wedge HTO for MMPRT in knees with moderate varus alignment. Third, two surgical techniques, namely, medial open-wedge HTO and medial open-wedge DTO, were used. However, it is well-known that there is no difference in clinical outcomes between medial open-wedge HTO and medial open-wedge DTO [5]. The strength of DTO is that it prevents distalization of the tuberosity [2, 5]. However, patients with symptomatic patellofemoral joint problems were excluded in this study to minimize the problem of multiple surgical procedures being performed in a short period. Finally, the difference in preoperative mean HKA angle between the moderate varus alignment group and the varus alignment group was relatively small. This may have been influenced by the indications for medial open-wedge HTO and medial open-wedge DTO at our institution. In other words, closed wedge HTO or other procedures may have been selected in cases of severe varus knee alignment.

## Conclusion

Favorable clinical results were obtained in patients with MMPRT and moderate varus knee alignment treated by medial open-wedge HTO. Surgeons should carefully consider the indications for medial open-wedge HTO even in knees with moderate varus alignment when planning treatment for MMPRT in patients with persistent knee pain.

## Supplementary Information

**Additional file 1.**

## Data Availability

The data that support the findings of this study are available from the corresponding author upon reasonable request.
